# Negative excess oral and pharyngeal cancer mortality in Europe during the early pandemic years

**DOI:** 10.1111/odi.15055

**Published:** 2024-06-27

**Authors:** Stefano Petti

**Affiliations:** ^1^ Department of Public Health and Infectious Diseases Sapienza University Rome Italy

**Keywords:** COVID‐19, excess mortality, mortality rate, oral cancer, pharyngeal cancer

## Abstract

**Objective:**

The COVID‐19 pandemic had direct and indirect effects on oral and pharyngeal cancer (OPC) mortality due to high COVID‐19 mortality risk among cancer patients, and to the COVID‐19 response that caused treatment delays and reduced routine visits. This study investigated the excess OPC mortality in Europe during the early pandemic years.

**Methods:**

Mortality and population data were gathered from the Eurostat database. The 2011–2019 mortality rates were used to estimate the 2020–2021 expected rates through joinpoint trend analysis. The excess mortality rates (observed minus expected mortality) with 95% confidence intervals (95 CIs) were assessed.

**Results:**

Statistically significant negative excess age‐standardized and crude (age strata <65 and ≥65 years) OPC mortality rates in males and females, in the European Union (EU, 27 countries) and Europe were reported. The estimated OPC missing deaths in EU were 831 (95 CI, 630–985) and 1240 (95 CI, 1039–1394) in 2020 and 2021, respectively, with differences between sexes, age strata, and countries. The OPC deaths in the EU and Europe were 3.6% and 3.5% lower than expected.

**Conclusion:**

Missing OPC deaths reported in Europe in 2020–2021 could be explained by changes in death certification of OPC patients who developed COVID‐19, rather than a real OPC mortality decline.

## 1 INTRODUCTION

The COVID‐19 pandemic posed a number of challenges to oral and pharyngeal cancer (OPC) patients, as cancer patients were at higher risk of COVID‐19 mortality (Dessie & Zewotir, 2021; Tagliamento et al., 2021). The pandemic also had an indirect impact on these subjects. Indeed, several standard treatment modalities were disrupted and non‐emergency visits were postponed due to the implementation of unprecedented public health measures aimed at mitigating SARS‐CoV‐2 transmission, and mobilizing the healthcare workforces for the COVID‐19 response. In addition, several cancer patients avoided seeking healthcare for non‐COVID‐19 related issues due to concerns about exposure to the virus (see, for example, da Cunha et al., 2021; Neamţiu et al., 2022, Rini & D'Urso, 2022). In April 2020, the World Health Organization (WHO) Regional Office for Europe recognized the impact of the COVID‐19 response on the healthcare sector, anticipating events, such as increased mortality, exacerbation of existing conditions, medical errors, mismanagement of conditions, and poorer quality of care (World Health Organization Regional Office for Europe, 2020a), and warned the European public health community that the continuity of essential services was warranted, while freeing up the resources to manage the COVID‐19 emergency (World Health Organization Regional Office for Europe, 2020b).

Prepandemic data provided by the European Community Statistical Office (Eurostat) showed that OPC mortality was declining in males, and increasing in females, thus, the number of saved deaths in males was counterbalanced by the number of extra deaths in females (Eurostat, 2024). However, this trend was expected to change in the early pandemic years, due to the direct and indirect impact of COVID‐19 pandemic on OPC patients. Indeed, according to the European Cancer Information System (ECIS), increases in cancer incidence and mortality during the COVID‐19 emergency were expected due to reduced diagnostic capacity and delayed referral (De Angelis et al., 2024), as already reported in other countries, such as Brazil (da Cunha et al., 2021, 2022), and the European Parliamentary Question “EU Action on Oral Cancer” asked for the inclusion of oral healthcare in the “Europe's Beating Cancer Plan” (European Parliament, 2021).

Surveys from healthcare settings reported various scenarios that could be summarized with treatment delays, a general decrease in routine visits, higher proportion of visited patients with advanced OPC stage, and stable number of deaths at the hospital level. Other surveys, however, reported data that were in conflict with these scenarios. For example, while the prolongation of patient delay was almost constantly reported, data on professional delay were varying, namely, prolonged in a University Hospital in Naples (Italy) and reduced in another University hospital in Montreal (Canada). The number of new diagnoses of oral squamous cell carcinoma generally decreased, as in the University Hospital in Turin (Italy), but increased in the University Hospital in Graz (Austria) (Alves et al., 2021; Arduino et al., 2021; Cwintal et al., 2024; da Cunha et al., 2022; D'Ascanio et al., 2022; Koyama et al., 2024; Lo Giudice et al., 2023; Machado et al., 2022; Metzger et al., 2021; Remschmidt et al., 2024; Tasoulas et al., 2023).

Since reliable conclusions on OPC mortality changes during the early pandemic years could not be drawn from the surveys from the field, the aim of this study was to investigate the excess OPC mortality in 2020 and 2021.

## 2 METHODS

### 2.1 Study design

OPC mortality data of all the European countries available from the Eurostat database were gathered. Mortality rates between 2011 (the oldest available data) and 2019 (the last prepandemic year) were used to estimate the rates expected to occur in the pandemic years 2020 and 2021 without the pandemic. The expected mortality was compared with the observed mortality to estimate the excess mortality.

### 2.2 Data extraction

The number of OPC deaths (ICD‐10 codes, C00‐C14) split for age, age‐standardized mortality rates (ASMRs) for OPC, and population from 2011 to 2021 were extracted from the Eurostat database. All the material used is freely available (Eurostat, 2024).

### 2.3 Excess oral and pharyngeal cancer mortality rate estimate

For each year and country, male and female crude OPC mortality rates were assessed. Crude mortality was chosen as an expression of the real‐life mortality and population losses due to OPC (Nepomuceno et al., 2022), and was also used to estimate the number of excess OPC deaths. Crude mortality was assessed for two age strata, <65 and ≥65 years. ASMRs also were considered, as ASMR is generally used to compare mortality trends across different countries with different population structures (e.g., Santucci et al., 2024).

The expected mortality rates for the years 2020 and 2021 were estimated with the joinpoint regression analysis using the Joinpoint Trend Analysis Software (Siegel et al., 2024). This regression model assumes that the regression function is piecewise linear and the segments are connected at trend‐change points, called joinpoints (Kim et al., 2014). The mortality trend was linearized starting from the oldest data, and when changes in trend occurred and the further data fit poorly the regression line, a new linearization with a different direction was made, starting from the joinpoint, that indicated the year when the trend change occurred. Extrapolations of the years 2020 and 2021 to estimate the expected mortality rates were made using the last segment, referring to the most recent prepandemic years. The standard error of the linear regression coefficient was used to assess the 95% confidence interval (95 CI) of the expected mortality. Mortality rates were logarithmically transformed to normalize the variance, thus smoothing the annual fluctuations. More details on cancer mortality prediction methods with the joinpoint regression are available (Chen et al., 2012; Zhu et al., 2012).

The excess OPC mortality rate (Nomura et al., 2023) was estimated with the formula:

$$\begin{array}{c} \begin{array}{c} \text{Excess}\;\text{mortality}\;\text{rate}=\left(\text{observed}\;\text{mortality}\;\text{rate}\right)\\ \;-\left(\text{expected}\;\text{mortality}\;\text{rate}\right) \end{array} \end{array}$$

Excess mortality could yield negative values, denoting that the observed mortality was lower than expected (i.e., missing mortality). The excess mortality 95 CI was assessed as the difference between the observed mortality (observed mortality has not the 95 CI, as it is not an estimate but a real data), and the lowest and the highest limits of the expected mortality 95 CI. And 95 CIs that did not include the “0” value were statistically significant, with *p* < 0.05.

The number of excess/missing OPC deaths with 95 CIs also was estimated with the formula:

$$\begin{array}{c} \text{Excess}\;\text{deaths}=\left[\left(\text{crude}\;\text{excess}\;\text{mortality}\;\text{rate}\right)\times \left(\text{population}\right)\right]/100,000 \end{array}$$

The 95 CI of the crude excess mortality rate was used to estimate the 95 CI of the number of excess deaths. The point estimates of the number of excess deaths were summed in males and females, in 2020 and 2021, to obtain the overall point estimated number of excess deaths, as for the overall 95 CIs, the lowest 95 CI limits were summed and the same was done with the highest 95 CI limits.

The analyses were made for males and females, for crude (age strata, <65 and ≥65 years) and age‐standardized mortality rates, for the European Union (EU; 27 countries) and all the European countries available from Eurostat (the 27 EU countries and Switzerland, Lichtenstein, Norway, Iceland, Serbia – improperly called Europe in this study) together.

### 2.4 Sensitivity analysis

Sensitivity analysis was made to investigate the reliability of the method used to estimate the excess mortality. Since the observed mortality did not change, the object of this analysis was the expected mortality, that was assessed using the following methods: (i) mortality rate in 2019, the last prepandemic year; (ii) average 2015–2019 mortality rate, the last five prepandemic years; (iii) 9‐year (2011–2019) linear regression; (iv) 9‐year (2011–2019) polynomial regression of order 2 (higher orders were not considered to prevent overfitting); (v) 5‐year median smoothing, based on median values for 5‐year periods (from 2011–2015 to 2015–2019) and linear regression; (vi) 5‐year (2015–2019) Poisson regression.

Crude mortality rates in females aged <65 years from the fifteen most populated countries were considered and the expected mortality rates in the year 2020 were assessed with the seven methods. This age‐sex group was chosen for the sensitivity analysis because it was the group with the lowest OPC mortality rate and, therefore, with the least predictable expected mortality.

The correlation between the expected rates estimated with these methods and the joinpoint regression analysis was assessed using both parametric Pearson's “r,” and nonparametric Spearman's “ρ.” Statistically significant correlations with *p* < 0.05 and coefficients higher than 0.5 were considered adequate.

### 2.5 Subgroup analysis

In order to investigate whether the estimated excess mortality in EU and Europe was influenced by the data from specific countries, the excess ASMR for the year 2021 was estimated in the five most populated countries.

The analyses were made using MedCalc 22.021 (MedCalc Software Ltd), StatView 5.0.1 (SAS Institute), Joinpoint Trend Analysis Software 5.1.0 (National Cancer Institute).

## 3 RESULTS

Data and trends of OPC ASMRs, crude mortality rates, and number of deaths are freely available from the Eurostat database, thus, they were not reported here (Eurostat, 2024). ASMR and crude mortality rates in males aged <65 years declined regularly in both the EU and Europe, while increased in males aged ≥65 years, with a peak in 2018, followed by a decline (Figure S1). Female ASMR reached a peak in 2019 and then declined, crude rates in females aged <65 years were steady in both EU and Europe then declined from 2019, while crude rates in females aged ≥65 years increased from 2011 to 2021. Overall, the number of OPC deaths in the EU was roughly 27,100 in 2011, peaked to 28,600 in 2018, and then declined to 27,500 in 2021.

Statistically nonsignificant male excess ASMR in the EU was found, namely, −0.06 per 100,000 individuals per year in 2020 and −0.11 in 2021 (Table 1). Negative crude excess mortality rates in the EU also were found, that were statistically significant, particularly in subjects aged ≥65 years. The estimated numbers of missing deaths in this age–sex class were 417 (95 CI, 357–487) and 482 (95 CI, 420–547) in 2020 and 2021, respectively. The situation in Europe was similar, the global number of missing deaths in males was 445 (95 CI, 262–582) in 2020 and 901 (95 CI, 418–1009) in 2021.

**TABLE 1:** Observed, expected, excess mortality rates for oral and pharyngeal cancer in the European Union (EU; 27 countries) and Europe (EU countries and Norway, Iceland, Switzerland, Lichtenstein, Serbia) in 2020 and 2021. Males.

	Observed rate	Expected rate	Excess rate	Excess deaths
Year 2020 – EU				
ASMR	9.58	9.64 (9.40; 9.76)	−0.06 (−0.18; 0.18)	
Crude rate (<65 years)	5.18	5.20 (5.15; 5.22)	−0.02 (−0.04; 0.03)	−29 (−66; 61)
Crude rate (≥65 years)	28.90	29.96 (29.81; 30.12)	−1.06 (−1.22; −0.90)*	−417 (−481; −357)*
Year 2021 – EU				
ASMR	9.34	9.45 (9.22; 9.58)	−0.11 (−0.24; 0.12)	
Crude rate (<65 years)	4.86	5.04 (4.99; 5.06)	−0.18 (−0.20; −0.14)*	−330 (−366; −243)*
Crude rate (≥65 years)	28.99	30.19 (30.04; 30.36)	−1.20 (−1.36; −1.05)*	−482 (−547; −420)*
Year 2020 – Europe				
Crude rate (<65 years)	5.11	5.11 (5.06; 5.13)	0.00 (−0.02; 0.05)	−4 (−43; 88)
Crude rate (≥65 years)	28.89	29.96 (29.74; 30.20)	−1.07 (−1.30; −0.85)*	−441 (−539; −350)*
Year 2021 – Europe				
Crude rate (<65 years)	4.77	4.96 (4.91; 4.98)	−0.19 (−0.21; −0.14)*	−346 (−354; −256)*
Crude rate (≥65 years)	28.88	30.21 (29.98; 30.44)	−1.32 (−1.56; −1.10)*	−555 (−655; −462)*

*Note*: ASMR for Europe was not provided by Eurostat. Mortality rates are expressed as number of deaths × 100,000 individuals per year, excess deaths are in numbers. 95% confidence intervals between brackets.

Abbreviation: ASMR, age‐standardized mortality rate.

* *p* < 0.05.

Female excess ASMR in EU was −0.14 per 100,000 individuals per year in both 2020 and 2021 and was statistically significant (Table 2). Similarly, statistically significant negative excess crude mortality rates in both the EU and Europe, in subjects aged <65 and ≥65 years, in 2020 and 2021 were found. The estimated global numbers of missing female deaths for all age classes in Europe were 374 (95 CI, 328–424) in 2020 and 385 (95 CI, 339–434) in 2021.

**TABLE 2:** Observed, expected, excess mortality for oral and pharyngeal cancer in the European Union (EU; 27 countries) and Europe (32 countries, namely, EU countries and Norway, Iceland, Switzerland, Lichtenstein, Serbia) in 2020 and 2021. Females.

	Observed rate	Expected rate	Excess rate	Excess deaths
Year 2020 – EU				
ASMR	2.63	2.77 (2.76; 2.78)	−0.14 (−0.15; −0.13)*	
Crude rate (<65 years)	1.13	1.24 (1.23; 1.26)	−0.11 (−0.13; −0.10)*	−199 (−226; −173)*
Crude rate (≥65 years)	9.83	10.18 (10.13; 10.23)	−0.35 (−0.41; −0.31)*	−186 (−212; −161)*
Year 2021 – EU				
ASMR	2.65	2.79 (2.79; 2.80)	−0.14 (−0.15; −0.13)*	
Crude rate (<65 years)	1.11	1.24 (1.22; 1.25)	−0.13 (−0.11; −0.12)*	−230 (−256; −204)*
Crude rate (≥65 years)	10.02	10.39 (10.34; 10.44)	−0.37 (−0.42; −0.32)*	−198 (−225; −172)*
Year 2020 – Europe				
Crude rate (<65 years)	1.12	1.23 (1.22; 1.24)	−0.11 (−0.12; −0.09)*	−198 (−226; −172)*
Crude rate (≥65 years)	9.86	10.18 (10.15; 10.22)	−0.32 (−0.36; −0.29)*	−176 (−198; −156)*
Year 2021 – Europe				
Crude rate (<65 years)	1.10	1.22 (1.21; 1.24)	−0.13 (−0.14; −0.11)*	−234 (−261; −208)*
Crude rate (≥65 years)	10.11	10.38 (10.35; 10.42)	−0.27 (−0.31; −0.24)*	−151 (−173; −131)*

*Note*: ASMR for Europe (32 countries) was not provided by Eurostat. Mortality rates are expressed as number of deaths × 100,000 individuals per year, excess deaths are in numbers. 95% confidence intervals between brackets.

Abbreviation: ASMR, age‐standardized mortality rate.

* *p* < 0.05.

The overall (males and females) estimated number of missing deaths in the EU was 831 in 2020 (95 CI, 630–985) and 1240 in 2021 (95 CI, 1039–1394). Therefore, the observed 55,414 deaths were 3.6% lower than expected. Missing deaths in Europe were 819 (95 CI, 590–1006) and 1286 (95 CI, 1057–1443) in 2020 and 2021. Therefore, the reported 57,659 deaths were 3.5% lower than expected.

The sensitivity analysis showed that in females younger than 65 years, the 2020 excess crude mortality estimates provided by the six investigated methods and the joinpoint regression were significantly correlated (Table S1). High correlation, with parametric and nonparametric correlation coefficients >0.9, between the joinpoint regression method and 2019 mortality, 2015–2019 average mortality, 2011–2019 polynomial regression, and 2015–2019 Poisson regression methods, were found.

The subgroup analysis included Germany, France, Italy, Spain, and Poland, and showed that there were differences between countries (Table S2). Namely, statistically significant positive excess ASMR was found in Spain in males and females, in 2020 and 2021; positive excess ASMR also was found in Germany, was statistically significant in males, but not in females; positive excess ASMR was reported in Poland, excluding for males in 2020; statistically significant negative excess mortality was found in France, excluding males in 2020, and in Italy.

## 4 DISCUSSION

Overall, negative excess OPC mortality in Europe in the early pandemic years was found, that was higher in 2021 than in 2020, with differences between sexes, age groups, and countries (Tables 1 and 2, Table S2). The global number of missing deaths was relatively small and accounted for roughly 2000 deaths, corresponding to less than 4% of all OPC deaths that occurred in this period. These data were consistent with the trends showing that the OPC mortality rates were almost consistently lower in 2020–2021 with respect to the latest prepandemic years (Figure S1).

Data on OPC deaths released by the Eurostat are collected by the national statistical institutes, and are based on the death certificates, these are not estimates, provisional data, or forecasts, but are real data. These data are periodically revised and validated by the national statistical institutes and the Eurostat (more information at, https://ec.europa.eu/eurostat/cache/metadata/en/demo_mor_esms.htm#data_rev1708508116751). Mortality rates from other organizations, such as the WHO mortality database, the Global Burden of Disease, and from the Eurostat before 2011, could be used to extend the present analysis to other countries outside Europe and for longer periods. However, since mortality rates reported by these organizations were based on both real and estimated data at the same time, yielded an unpredictable level of uncertainty (see, for example, De Angelis et al., 2024; GBD 2019 Lip, Oral, and Pharyngeal Cancer Collaborators, 2023; Santucci et al., 2024). Therefore, they were not considered comparable enough with the 2011–2021 Eurostat data, that acknowledged the Quality Assurance Framework (https://ec.europa.eu/eurostat/web/quality). The use of different databases with different levels of reliability and quality results in incomparable rates between countries, as shown by the excess all‐cause mortality during the pandemic (Kelly et al., 2021).

The assessment of excess mortality at the population level implies that a real variable, the observed mortality, is compared with a fictional variable, the expected mortality, that cannot be verified. Thus, acknowledging the reliability of the observed mortality, the potential limit of this study was the uncertainty regarding the expected mortality.

Studies investigating the effect of cancer risk factors/preventive measures (e.g., Eloranta et al., 2012; Fernández‐Navarro et al., 2017) estimate the expected mortality comparing the rate in the group exposed to the risk/preventive factor, with the rate in the unexposed group. The latter is considered the mortality that would be observed in the exposed group without the investigated factor. In contrast, in population‐based studies there are no comparators, and the expected mortality is estimated using the mortality of previous years. Usually, mortality projections are considered accurate when the difference between the projected and the observed mortality rates is minimal (Lever et al., 2016). However, such evaluation was impossible, because the pandemic had direct and indirect effects on non‐COVID‐19 mortality, as predicted by the WHO, and the discrepancy between expected and observed mortality rates was anticipated (World Health Organization, 2021).

There are many methods to estimate the expected mortality, are all limited. The 5‐year average mortality and the last‐year mortality rates are valid as far as mortality rates are stable, while increasing and decreasing trends produce under‐ and overestimates of excess mortality, respectively. The linear regression method accounts for increasing/decreasing trends, but does not account for trend changes, and therefore, is valid as far as linearization of mortality rates is possible (see, for example, De Angelis et al., 2024). Complex methods such as nonlinear polynomial regressions, splines, and multivariable models account for trend changes. Trend changes, however, could be true or apparent. The latter are casual fluctuations and are usually due to technical problems, such as misreporting, misreporting corrections made in following years, and modifications of the reporting and the surveillance systems. The main problem of these methods is that they could fail to distinguish between fluctuations and real trend changes, because they adapt excessively to the training data and perform poorly with the new data, as happened to the exaggerated excess all‐cause mortality estimated in Germany by the WHO during the pandemic (Ferenci, 2023; Lever et al., 2016; Sanchez & Marzban, 2020; van Noorden, 2022).

Smoothing techniques seek to reduce the impact of casual fluctuations. Indeed, Poisson and quasi‐Poisson regressions account for fluctuations thanks to the logarithmic transformation of mortality rates. Poisson regression was used during the COVID‐19 pandemic to estimate the excess number of all‐cause deaths (Barnard et al., 2022), and the excess OPC incidence (Peacock et al., 2023). However, these methods imply that data are dispersed (Poisson distribution) or overdispersed (quasi‐Poisson distribution) (Barnard et al., 2022). These requisites were not satisfied by the observed OPC mortality rates. Another smoothing technique is the average/median‐smoothing method, originally developed to estimate the excess influenza/pneumonia mortality. Instead of considering the actual mortality rates for each year, this method considers the averages or the medians of the mortality rates of the adjacent years (Collins et al., 1930). This smoothing technique is widely used to assess disease trends when fluctuations are large, such as the 7‐day smoothing used for daily COVID‐19 mortality trends (Our World in Data, 2024), and the 3‐year smoothing used as sensitivity analysis by the EUROCARE‐6 for the 2020 cancer prevalence projections (De Angelis et al., 2024). Smoothing techniques reduce data dispersion, thus allowing linearization, but do not account for trend changes.

OPC mortality trends show considerable annual variability, possibly due to both trend changes and fluctuations (GBD 2019 Lip, Oral, and Pharyngeal Cancer Collaborators, 2023). For this reason, the joinpoint regression analysis, developed by the US National Cancer Institute, was used in this study. This method accounted for trends, through linear regression analysis, trend changes, through the piecewise linear function, and fluctuations, through logarithmical transformation of the dependent variable (Siegel et al., 2024) and proved to be effective, although has reduced power to account for fluctuations due to misreporting (Chen et al., 2012; Miller et al., 2021).

To investigate the validity of the reported results, the number of OPC deaths for the year 2022 estimated with the present method was compared with the estimate made by the European Cancer Information System, based on linear trends (ECIS, 2024). The estimated OPC deaths were 29,430 and 28,715 according to the ECIS, and the joinpoint regression analysis, respectively (data not in Table), with a difference between methods of just 2.4%, that corroborated the results of the present study. Therefore, relatively robust conclusions regarding the negative excess mortality could be drawn, although uncertainty remained, due to the ecologic design of the present study that evaluated nation‐based instead of individual‐based data and prevented a straightforward analysis of the causes of the reported excess or missing OPC deaths (Petti & Cowling, 2020).

Although the excess OPC mortality was globally negative in Europe, there were differences between the five most populated countries (Table S2), with Italy and France that showed negative excess mortality, Spain and Poland that showed positive excess mortality, and Germany that showed positive excess mortality in males and negative in females. Positive excess mortality for chronic diseases was predicted by the WHO Regional Office for Europe from April 2020, due to the likely disruption of the healthcare systems (World Health Organization Regional Office for Europe, 2020a, 2020b), that actually occurred in several European countries. In addition, cancer patients were advised not to visit hospitals unless under very specific circumstances, and fear of developing SARS‐CoV‐2 in healthcare settings further reduced the number of cancer‐related visits (Muka et al., 2023; Muls et al., 2022). If positive excess OPC mortality was somewhat conjectured, negative mortality was not.

To explain such an apparently puzzling result, the hypothesis that new and more effective OPC treatments were implemented during the early pandemic years in Europe is not plausible, since the oral cancer survival rate in Europe (De Angelis et al., 2024) is considerably lower than in the US (National Institute of Dental and Craniofacial Research, 2023). Misestimates could also be implicated. However, there could be other and more plausible explanations. The US Centers for Disease Control and Prevention investigated cancer mortality in pre‐ and early pandemic years, analyzing the death certificates. They reported that the overall number of certificates with OPC as underlying (i.e., principal) or contributing (i.e., comorbidity) cause of death actually increased from 2019 to 2020 by 5%, as expected, but the number of certificates with OPC as underlying cause of death was stable, thus giving the wrong impression that OPC mortality did not change substantially during the first pandemic year. Such discrepancy was explained by the high number of certificates that had COVID‐19 as the principal cause of death and OPC as comorbidity, particularly among older persons, males, and certain ethnic groups. Indeed, the proportion of death certificates with OPC as the principal cause of death dropped from 89% in 2018–2019 to 87% in 2020 and 86% in 2021 (Henley et al., 2022). Thus, the missing OPC deaths reported in Europe in 2020–2021 could be not due to a real decline in mortality, that would be inexplicable during the early pandemic years, but to changes in how the death certificates of OPC patients with COVID‐19 were written. This hypothesis was corroborated by the guidelines for the certification and classification of COVID‐19 as the cause of death, released by the WHO in April 2020 (World Health Organization, 2020). According to this document, “COVID‐19 should be recorded on the medical certificate of cause of death for all decedents where the disease caused, or is assumed to have caused, or contributed to death” and “if the decedent had existing chronic conditions… they should be reported in Part‐2 of the medical certificate” (i.e., as comorbidities). Thus, starting from April 2020, chronic conditions, such as cancer were classified as contributing causes of death in several subjects with COVID‐19. Indeed, cancer appeared as comorbidity in as many as 17% COVID‐19 death certificates in 2020 in Italy (Grippo et al., 2021). The contribution of cancer as the cause of death in patients with COVID‐19 was underreported in the early pandemic years, as reported by the US COVID‐19 Surveillance System, that found that cancer was listed as comorbidity in only 4% of certificates in 2020, while comparing these certificates with the information gathered from the National Electronic Disease Surveillance System, the real proportion was 10% (Parker et al., 2021).

These results may help reconcile the OPC mortality data with surveys from the field that reported general decrease in routine visits, treatment delays, and higher proportion of OPC patients presenting with advanced cancer stage during the early pandemic years, suggesting higher mortality in OPC patients (Alves et al., 2021; Arduino et al., 2021; Cwintal et al., 2024; da Cunha et al., 2022; D'Ascanio et al., 2022; Koyama et al., 2024; Lo Giudice et al., 2023; Machado et al., 2022; Metzger et al., 2021; Remschmidt et al., 2024; Tasoulas et al., 2023). Indeed, OPC mortality could actually increase in 2020–2021 in Europe, but could apparently decrease, because in an unknown proportion of deaths among OPC patients with COVID‐19, because OPC was classified as comorbidity or was even excluded from the certificate. In order to explain this negative excess mortality, further studies comparing the death certificates with the hospital files are needed in the European context, to investigate the causes of this apparently puzzling phenomenon and its reliability.

## AUTHOR CONTRIBUTIONS

**Stefano Petti:** Conceptualization; investigation; writing – original draft; methodology; validation; visualization; writing – review and editing; software; formal analysis; data curation.

## CONFLICT OF INTEREST STATEMENT

The author declares that he has no competing financial interests or personal relationships that could influence the work reported in this paper.

## Supporting information


Appendix S1.

## Data Availability

The data that support the findings of this study are freely available from the Eurostat Data Browser: https://ec.europa.eu/eurostat/databrowser/explore/all/all_themes.
