# Supplementary material for: Negative excess oral and pharyngeal cancer mortality in Europe during the early pandemic years
Source: Oral Dis. 2024 Jun 27;31(1):121–8. doi: 10.1111/odi.15055 (PMC11808167; doi:10.1111/odi.15055)
Supplement: Supplementary file 1 — Appendix S1. [file ODI-31-121-s001.pdf]

Negative excess oral and pharyngeal cancer mortality in Europe during the pandemic years 2020 and 2021

Stefano Petti [1]

[1] Department of Public Health and Infectious Diseases, Sapienza University, Rome, Italy

SUPPLEMENTARY MATERIAL

FIGURE S1 – 2011-2021 trends in age-standardized (ASMR) and crude (age strata, <65 years, ≥65 years) oral and pharyngeal cancer mortality rates in EU (27 countries) and Europe (EU countries and Switzerland, Lichtenstein, Norway, Iceland, Serbia), and polynomial regression curves of order 6. Mortality rates are expressed as number of deaths per 100,000 persons per year.

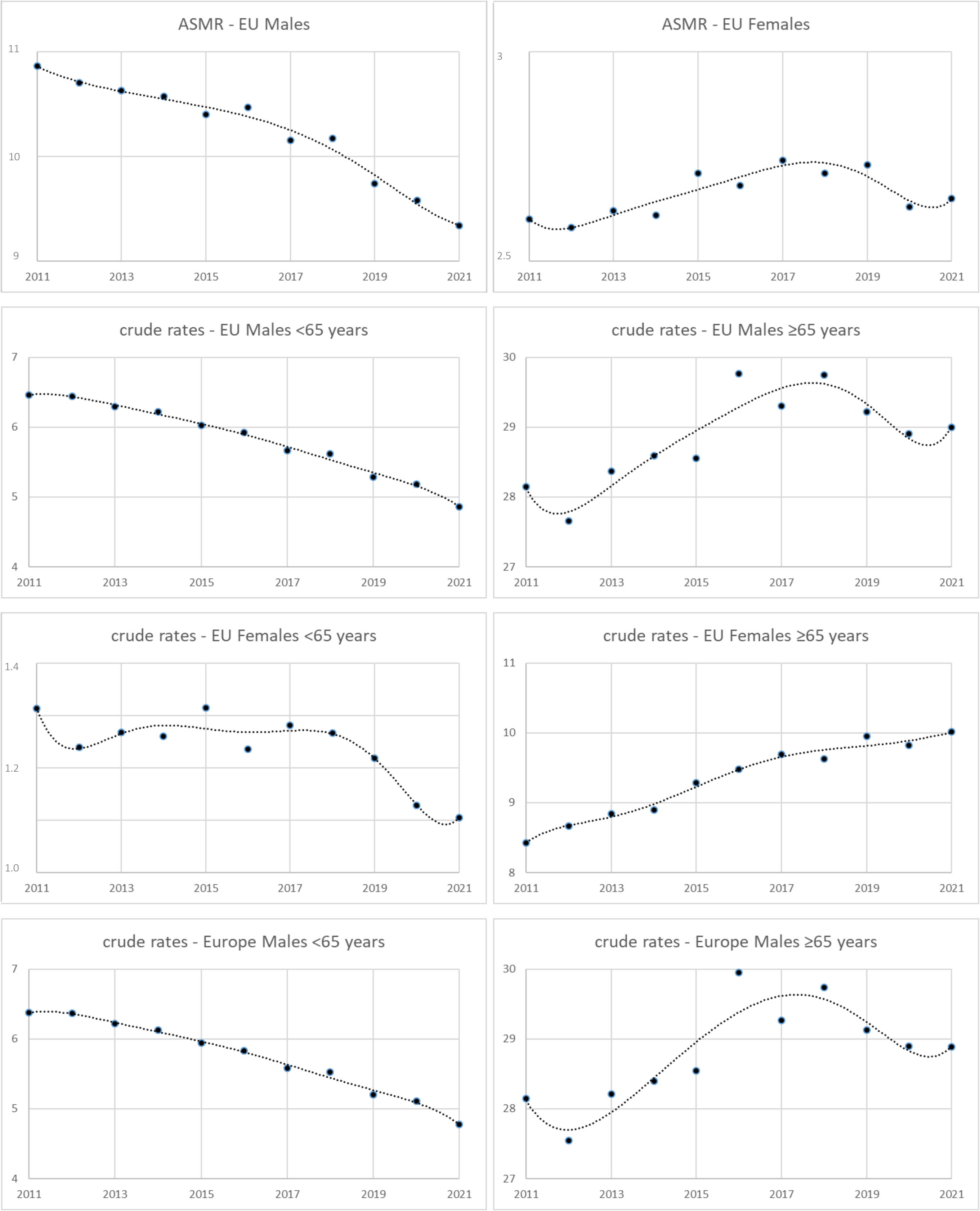

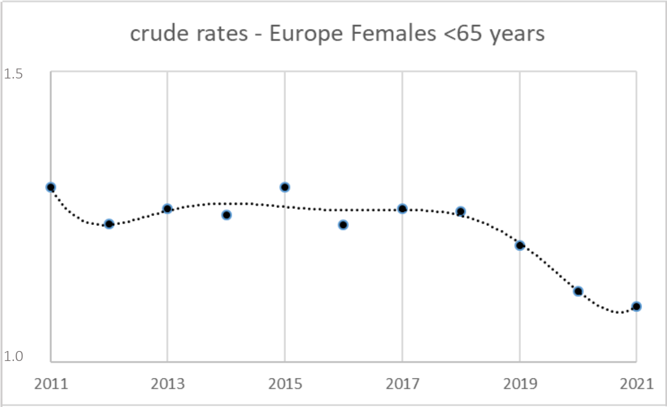
crude rates - Europe Females <65 years
1.5
1.0
2011
2013
2015
2017
2019
2021

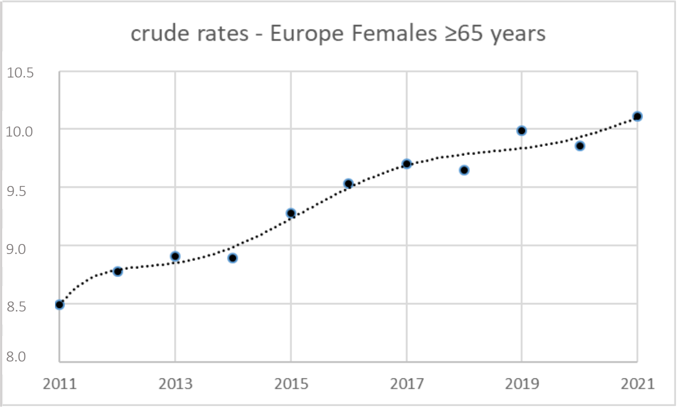
crude rates - Europe Females ≥65 years
10.5
10.0
9.5
9.0
8.5
8.0
2011
2013
2015
2017
2019
2021

TABLE S1 – Sensitivity analysis to the method chosen to estimate the expected mortality rates (crude mortality rates expected in 2020 in females aged <65 years) in the fifteen most populated European countries. Pearson's and Spearman's correlation coefficients between the chosen method (9-year joinpoint regression analysis) and other methods. Namely, mortality rate in the last prepandemic year, 2019; average mortality rate of the last five prepandemic years 2015-2019; 9-year (2011-2019) linear regression; 9-year (2011-2019) polynomial regression of order 2; 5-year median smoothing with linear regression; 5-year (2015-2019) Poisson regression.

Method	Pearson's r	Spearman's ρ
last-year rate	0.989	0.971
5-year average rate	0.983	0.961
2011-2019 linear regression	0.776	0.889
2011-2019 polynomial regression	0.980	0.985
5-year median smoothing	0.708	0.564
2015-2019 Poisson regression	0.995	0.996

All the correlations coefficient estimates were statistically significant, with $p<0.05$

Included countries, Germany, France, Italy, Spain, Poland, Romania, Netherlands, Belgium, Greece, Czechia, Sweden, Portugal, Hungary, Austria, Switzerland

TABLE S2. Observed, expected, excess age-standardized mortality rates (ASMRs) in 2020 and 2021 for oral and pharyngeal cancer in the five most populated European countries.

Country	Observed ASMR	Expected ASMR	Excess ASMR
		Germany	
Male 2020	9.15	8.86 (8.67; 8.95)	0.29 (0.20; 0.48)*
Male 2021	8.75	8.62 (8.43; 8.71)	0.13 (0.04; 0.32)*
Female 2020	2.66	2.82 (2.57; 3.07)	-0.16 (-0.41; 0.09)
Female 2021	2.69	2.82 (2.57; 3.07)	-0.13 (-0.38; 0.12)
		France	
Male 2020	8.74	8.53 (8.48; 8.59)	0.21 (0.15; 0.26)*
Male 2021	8.04	8.22 (8.17; 8.28)	-0.18 (-0.23; -0.13)*
Female 2020	2.25	2.32 (2.26; 2.37)	-0.07 (-0.12; -0.01)*
Female 2021	2.09	2.31 (2.25; 2.36)	-0.21 (-0.27; -0.16)*
		Italy	
Male 2020	6.43	6.90 (6.79; 7.02)	-0.47 (-0.58; -0.36)*
Male 2021	6.48	6.89 (6.78; 7.00)	-0.41 (-0.52; -0.30)*
Female 2020	2.47	2.64 (2.61; 2.68)	-0.17 (-0.21; -0.14)*
Female 2021	2.50	2.69 (2.65; 2.72)	-0.19 (-0.22; -0.15)*
		Spain	
Male 2020	7.64	7.21 (6.86; 7.55)	0.43 (0.09; 0.78)*
Male 2021	7.59	6.82 (6.48; 7.14)	0.77 (0.45; 1.11)*
Female 2020	2.40	2.30 (2.27; 2.33)	0.10 (0.07; 0.13)*
Female 2021	2.43	2.31 (2.28; 2.34)	0.12 (0.09; 0.15)*
		Poland	
Male 2020	13.64	13.77 (13.48; 14.19)	-0.13 (-0.65; 0.06)
Male 2021	13.91	13.60 (13.31; 14.01)	0.31 (-0.10; 0.60)
Female 2020	3.61	3.54 (3.29; 3.63)	0.07 (-0.02; 0.32)
Female 2021	3.73	3.57 (3.32; 3.63)	0.16 (0.10; 0.41)*

ASMRs are expressed as number of deaths x 100,000 individuals per year, 95% confidence intervals between brackets. *$p<0.05$
